# Asian sand dust exacerbates airway inflammation in a mouse model of asthma

**DOI:** 10.1186/s42826-025-00243-9

**Published:** 2025-05-09

**Authors:** Se-Jin Lee, So-Won Pak, Woong-Il Kim, Sin-Hyang Park, Young-Kwon Cho, Tae-Won Kim, Je-Won Ko, Joong-Sun Kim, Jong-Choon Kim, In-Hyeon Kim, Sung-Hwan Kim, In-Sik Shin

**Affiliations:** 1 College of Veterinary Medicine and BK21 FOUR Program, Chonnam National University, 77 Yong-bong-ro, Buk-gu, Gwangju, 61186 Republic of Korea; 2 College of Health Sciences, Cheongju University, 298 Daesung-ro, Sangdang-gu, Cheongju-si, Chungbuk, 28503 Republic of Korea; 3 BK21 FOUR Program, College of Veterinary Medicine, Chungnam National university, 99 Daehak-ro, Daejeon, 34134 Republic of Korea; 4 Jeonbuk Branch, Korea Institute of Toxicology (KIT), Jeongeup-si Jeonbuk, 53212 Republic of Korea

**Keywords:** Asian sand dust, Pulmonary toxicity, Allergic asthma, Nuclear factor-κappa B

## Abstract

**Background:**

Asian sand dust (ASD), generated from the deserts of China and Mongolia, mainly affects the human health of several countries in Northeast Asia including China, Korea, and Japan. In this study, we investigated the toxic effects of ASD on respiratory tract and explored the effects of ASD exposure on allergic asthma using ovalbumin-induced asthma model. C57BL/6 male mice were used for both the toxicity and allergic asthma studies. ASD (10, 20, and 40 mg/kg) was administered intranasally on days 1, 3, and 5. For allergic asthma, mice were sensitized with OVA (20 µg/mouse) and aluminum hydroxide (2 mg) on days 1 and 15, followed by OVA inhalation (1%, w/v) on days 22, 24, and 26, with subsequent ASD instillation on days 21, 23, and 25.

**Results:**

ASD exposure showed the elevation of respiratory inflammation including inflammatory cell infiltration, cytokine production, and mucus secretion with the increase in phosphorylated (p)-nuclear factor-kappa B (NF-κB) p65 expression. In addition, ASD exposure to asthma model significantly increased airway responsiveness, inflammatory cell count and mucus secretion with the elevation of cytokines and immunoglobulin E, which were accompanied with the increases in p-NF-κB p65, p-p38 and cyclooxygenase 2 (COX2).

**Conclusions:**

Therefore, ASD exposure induces respiratory inflammation and aggravates the progression of allergic asthma, which was closely associated with the phosphorylation of NF-κB. Respiratory exposure to ASD causes inflammation, upregulation of cytokines, p-NF-κB, and COX2, which can exacerbate asthma.

**Supplementary Information:**

The online version contains supplementary material available at 10.1186/s42826-025-00243-9.

## Background

Asian sand dust (ASD) is an air-pollutant from the deserts of China and Mongolia [1]. ASD is commonly called yellow dust or China dust, and mainly affects Northeast Asia including China, Korea, and Japan during the spring and autumn [2]. Additionally, since the number of patients with respiratory disease increases during these seasons due to temperature changes and decreased immunity, exposure to ASD may lead to an increase in patients with respiratory diseases and worsening of underlying diseases. Because the use of masks is recommended during these corresponding seasons, normal activities of people are complicated [3]. In addition, as desertification and industrialization progress, various harmful substances generated therefrom have been included in ASD, threatening human health further. According to a recent study, ASD has increased particulate matter (PM)10 and PM2.5 levels and includes PM-bound trace metals such as aluminum (Al), arsenic (As), calcium (Ca), copper (Cu), Iron (Fe), nickel (Ni), lead (Pb), and Zinc (Zn) and various bacteria, which increase the occurrence of cardiovascular and respiratory diseases [4]. Furthermore, because ASD contains a lot of silica, there is a risk of causing silicosis, which is proven by the occurence of silicosis in artificial stone workers who are at high risk of exposure to silica dust [5]. Therefore, ASD is regarded as an important social issue that threatens human health. This is not limited to Korea, as it is also considered an important social problem in China and Japan, which are affected by ASD via air currents [6, 7].

Allergic asthma is regarded as a complex inflammatory disorder of the pulmonary system that has features such as airway hypersensitivity, eosinophilia, and mucus production, leading to several clinical symptoms such as chest tightness, cough, dyspnea, and wheezing [8]. It is caused by exposure to allergens, which induce asthmatic characteristics via inflammatory cytokines such as interleukin (IL)-4, -5, and − 13 [9]. During the progression of allergic asthma, exposure to other exogenous stimuli exacerbates the symptoms of allergic asthma, and recently, as ASD has increased, the incidence of allergic asthma has increased and the condition of patients with allergic asthma has worsened [4].

Various signaling pathways related to inflammatory responses are associated with the pathogenesis of allergic asthma. Regarding signaling pathways, nuclear factor-kappa B (NF-κB) acts as a crucial transcription factor that produces various inflammatory mediators and is related to the pathogenesis of various diseases [10]. The most abundant form of NF-κB is the p50/p65 dimer, and the phosphorylation of p65 by pathologic stimuli plays a pivotal role in several chronic inflammatory conditions [11]. In the progression of allergic asthma, elevated phosphorylated (p)-NF-κB expression induces the releases of inflammatory mediators including cytokines, cyclooxygenase 2 (COX2) and matrix metalloproteinases, which eventually accelerate airway inflammation. In particular, tumor necrosis factor (TNF)-α and IL-6 induce acute inflammatory responses by participating in the recruitment and activation of inflammatory cells [12]. When respiratory system is exposed to specific irritants, the production of TNF-α and IL-6 increases and the resulting inflammatory response in lung tissue is significantly aggravated [13]. Moreover, in asthma, TNF-α interacts with other cytokines, leading to the recruitment of inflammatory cells to the airways and contributing to airway remodeling [14]. Thus, NF-κB is regarded as a major target for allergic asthma and an indicator of asthma severity [13].

Therefore, we investigated the pulmonary toxicity induced by ASD exposure and then evaluated its aggravative effect on allergic asthma caused by ovalbumin (OVA). Furthermore, to identify the toxic mechanism of ASD, we performed a biochemical analysis focusing on NF-κB.

## Methods

### Asian sand dust (ASD)

ASD (POWDER TECHNOLOGY Inc., Arden Hills, MN) is a 50/50 mixture of JIS Z8901 Class 8 test dust and naturally occurring silicon dioxide. The morphology of ASD was determined using transmission electron microscopy (TEM, JEM-2100 F, JEOL, Tokyo, Japan), and the primary size of ASD was evaluated using scanning electron microscopy (SEM, Zeiss Gemini500, Carl Zeiss Meditec AG, Jena, Germany), and subsequently measuring the sizes of 200 particles using ImageJ software. The purity of ASD was evaluated using energy-dispersive X-ray spectroscopy (Oxford Instrument, Abingdon, UK). Zeta potential and hydrodynamic size were measured using a dynamic light scattering instrument (ELSZneo, Otsuka Electronics, Tokyo, Japan).

### Experimental design

**Fig. 1:**
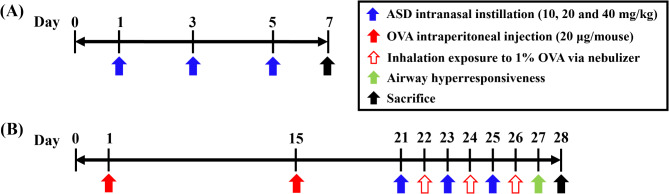
Experimental procedures of pulmonary toxicity and allergic asthma. **(A)** Experimental procedure for pulmonary toxicity, **(B)** experimental procedure for allergic asthma

C57BL/6 male mice (6 weeks old, 18–20 g) without specific pathogens were purchased from SAMTAKO (Osan, Republic of Korea). To establish a toxicity study of ASD, 28 animals were randomly designed into four experimental groups (each group, *n* = 7) as follows: NC (normal control), and ASD 10, 20, and 40 (10, 20, and 40 mg/kg of ASD, respectively) (Fig. 1A). ASD was administered by intranasal instillation on days 1, 3, and 5. Exposure dose of ASD was determined according to previous study [2]. The instillation dose of ASD (40 mg/kg) was 5.3 times higher than the national air quality standard for suspended PM, as PM levels during ASD events in Korea often exceed 150 µg/m³ [15].

To investigate the deteriorative effects of ASD exposure on allergic asthma, mice were designated into 5 groups (each group, *n* = 7) as follows: NC (normal control), OVA (asthma model), and ASD10, 20, and 40 + OVA (10, 20, and 40 mg/kg of ASD exposure + asthma model). As shown in Fig. 1B, the protocol to induce asthma was conducted according to previous studies [8, 12, 16]. The animals received OVA (A5503, Sigma-Aldrich, St. Louis, MO) sensitization (20 µg/mouse of OVA + 2 mg of aluminum hydroxide (77161, Thermo Fisher, San Diego, CA)) via intraperitoneal administration on days 1 and 15. OVA (1%, w/v, in phosphate-buffered saline (PBS)) inhalation was performed once a day for 1 h on days 22, 24, and 26 using a nebulizer (Omron, Tokyo, Japan). The intranasal instillation of ASD (10, 20, and 40 mg/kg) was performed once a day on days 21, 23, and 25. Airway hyperresponsiveness (AHR) was performed 24 h after the final OVA inhalation. AHR of the mice was assessed using single-frequency forced oscillation with Flexivent (SCIREQ, Montreal, Canada). Under anesthesia with alfaxalone (Jurox Pty Ltd., Hunter Valley, Australia), a tracheostomy was performed to insert a cannula connected to a nebulizer containing PBS or methacholine (0, 10, 20, and 40 mg/mL; A2251, Sigma-Aldrich). The sequence of making measurements, nebulizing with methacholine solution for 10 s, recording airway resistance for 1 min, and refreshing for 2 min, was automatically repeated.

The animals used in toxicity and asthma experiments were sacrificed on days 7 and 28, respectively. Bronchoalveolar lavage fluid (BALF) was collected 48 h after the final challenge. Mice were anesthetized via intraperitoneal injection of alfaxalone (Jurox Pty Ltd.) and underwent tracheostomy. The lungs were lavaged twice with 0.7 mL of ice-cold PBS, and the obtained BALF samples were then centrifuged. The separated supernatants were used to measure inflammatory cytokines. BALF pellets were resuspended with PBS and then the number of total inflammatory cells was measured by an automated cell counter (Thermo Fisher). In addition, resuspended BALF pellets were attached to glass slides using cytospin (Hanil Science Industrial, Seoul, Korea), which were stained with Diff-Quik solution (Sysmax, Kobe, Japan) and representative pictures per slide were captured by microscopy using a slide scanner (Motic, Hong Kong). We counted inflammatory cells including neutrophils, macrophages, eosinophils, and lymphocytes, which were applied to total inflammatory cell counts obtained from the cell counter to determine the number of differential inflammatory cell counts.

Approval of animal studies was in accordance with the ethical guidelines and regulations established by the Institutional Animal Care and Use Committee of Chonnam National University (IACUC) carried out under approval numbers CNU IACUC-YB-R-2020-93 and CNU IACUC-YB-R-2021-36.

### Analysis of pathophysiological mediators in BALF and serum

BALF supernatant was used to determine the release of inflammatory cytokines including TNF-α, IL-4, -6, and − 13 using commercial enzyme-linked immunosorbent assay (ELISA) kit (R&D Systems, Minneapolis, MN). Blood sample was draw from the caudal vena cava and centrifuged to collect serum, which was used to evaluate OVA-specific immunoglobulin (Ig) E using commercial ELISA kit (BioLegend, San Diego, CA).

### Histopathological examination

The left pulmonary lobes from each mouse were fixed with 10% neutralized formalin (Sigma-Aldrich) and embedded in paraffin to make a paraffin block for the evaluation of histological alterations. Then, paraffin blocks were cut to a 4 μm thickness and attached to glass slides. For the evaluation of histological alterations including inflammatory cell accumulation and mucus production, the slides were stained with hematoxylin and eosin (Sigma-Aldrich) or periodic acid Schiff’s solution (Sigma-Aldrich). Furthermore, to determine the expression of p-NF-κB p65 in pulmonary tissues, we performed immunohistochemistry using commercial kits (Vector Laboratories, Burlingame, CA). The primary antibody for p-NF-κB p65 (MA5-15160, Thermo Fisher) was diluted at a ratio of 1:200, and the procedure was conducted according to the protocol provided by the manufacturer. The representative figures for histological examination were obtained using a slide scanner (Motic) and the quantification of pulmonary inflammatory responses, mucus secretion, and protein expression were carried out using an IMT i-Solution, as an image analyzer (IMT i-Solution Inc., Vancouver, BC, Canada). In quantitative analysis of inflammation, we evaluated inflammatory response for total area (captured on 200 × magnification). In quantitative analysis of mucus secretion, we evaluated mucus secretion for bronchial area. Quantitative value was expressed as percent (%, inflammation or mucus secretion area vs. arranged area).

### Western blotting

Right pulmonary lobes were homogenized in a tissue lysis/extraction reagent (1/10 w/v; Sigma-Aldrich) contained with a protease inhibitor (P8340, Sigma-Aldrich). The protein quantification for each sample was performed using Bradford reagent (5000205, Bio-Rad, Hercules, CA) according to the manufacturer’s instructions. A total of 30 µg of cellular protein was quantified and then separated using electrophoresis. The separated proteins were transferred to a nitrocellulose membrane, followed by incubation in blocking solution (5% skim milk). After removing the blocking solution, the membrane was incubated with the primary antibody at 4 °C overnight. After removing the primary antibody, the membrane was washed three times with Tris-buffered saline containing Tween 20 (TBST). Following the removal of the Tris buffer, the membrane was incubated with the secondary antibody, goat anti-rabbit IgG (H + L) HRP (1:10,000 dilution; 31460, Thermo Fisher), at room temperature for 30 min. After removing the secondary antibody, the membrane was washed five times with TBST for 15 min each. The quantification of protein expression was performed using Chemi-Doc (Bio-Rad). The primary antibodies used were as follows: p-p38 (1:1000; 4511, Cell signaling, Danvers, MA), p-NF-κB p65 (1:1000; MA5-15160, Thermo Fisher), β-actin (1:1000; 4967, Cell Signaling), and COX2 (1:1000; ab15191, Abcam, Waltham, MA).

### Statistical analysis

The data are expressed as the mean ± standard deviation. Statistical analysis was performed using analysis of variance followed by Dunnett’s adjustment using GraphPad Prism 5 (GraphPad Software, CA). A p-value < 0.05 was regarded as statistically significant.

## Results

### Characteristic analysis of ASD

**Fig. 2:**
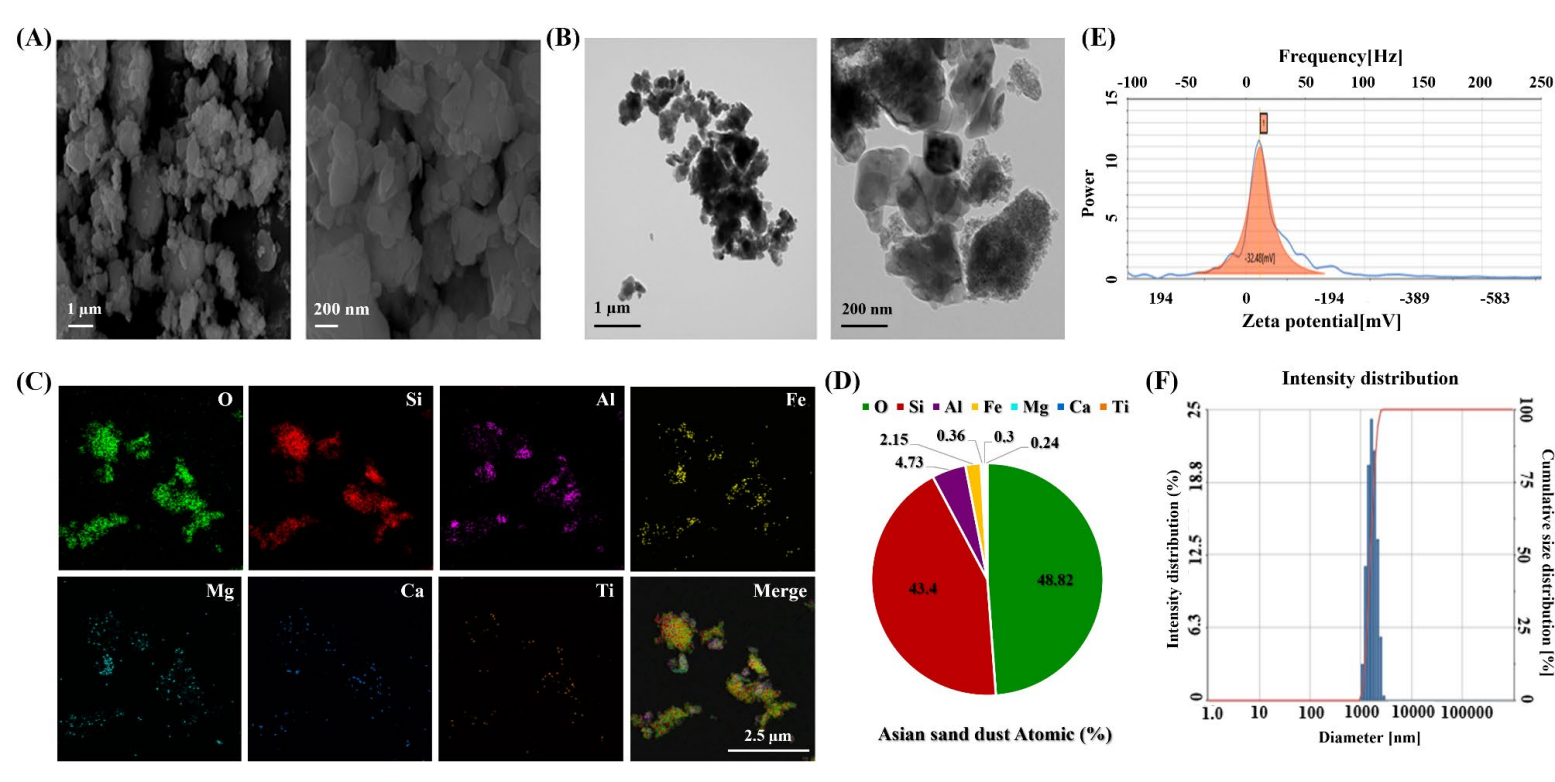
Characteristic of Asian Sand Dust (ASD). **(A)** Figure of Scanning electron microscopy (SEM) for ASD, **(B)** figure of Transmission electron microscopy (TEM) for ASD, **(C)** representative figure of ASD composition analyzed by X-ray spectroscopy on SEM images, **(D)** quantitative analysis of ASD composition, **(E)** average zeta potential of ASD in water, and **(F)** average particle size of ASD in water

The morphology and size of ASD were measured by SEM and TEM analysis (Fig. 2A, B). The component segments of ASD were investigated by TEM analysis (Fig. 2C). ASD morphology was mostly spherical and the primary size of ASD was 292 ± 186.2 nm. The composition of ASD consisted of 48.82% O, 43.4% Si, 4.73% Al, 2.15% Fe, 0.36% Mg, 0.3% Ti, and 0.24% Ca (Fig. 2D). As a result of measuring the zeta-potential of ASD, the average negative zeta potential was − 32.48 mV (Fig. 2E). The average diameter of ASD in water was 1807.2 nm (Fig. 2F).

### ASD exposure increased the inflammatory indices in the BALF of mice

**Fig. 3:**
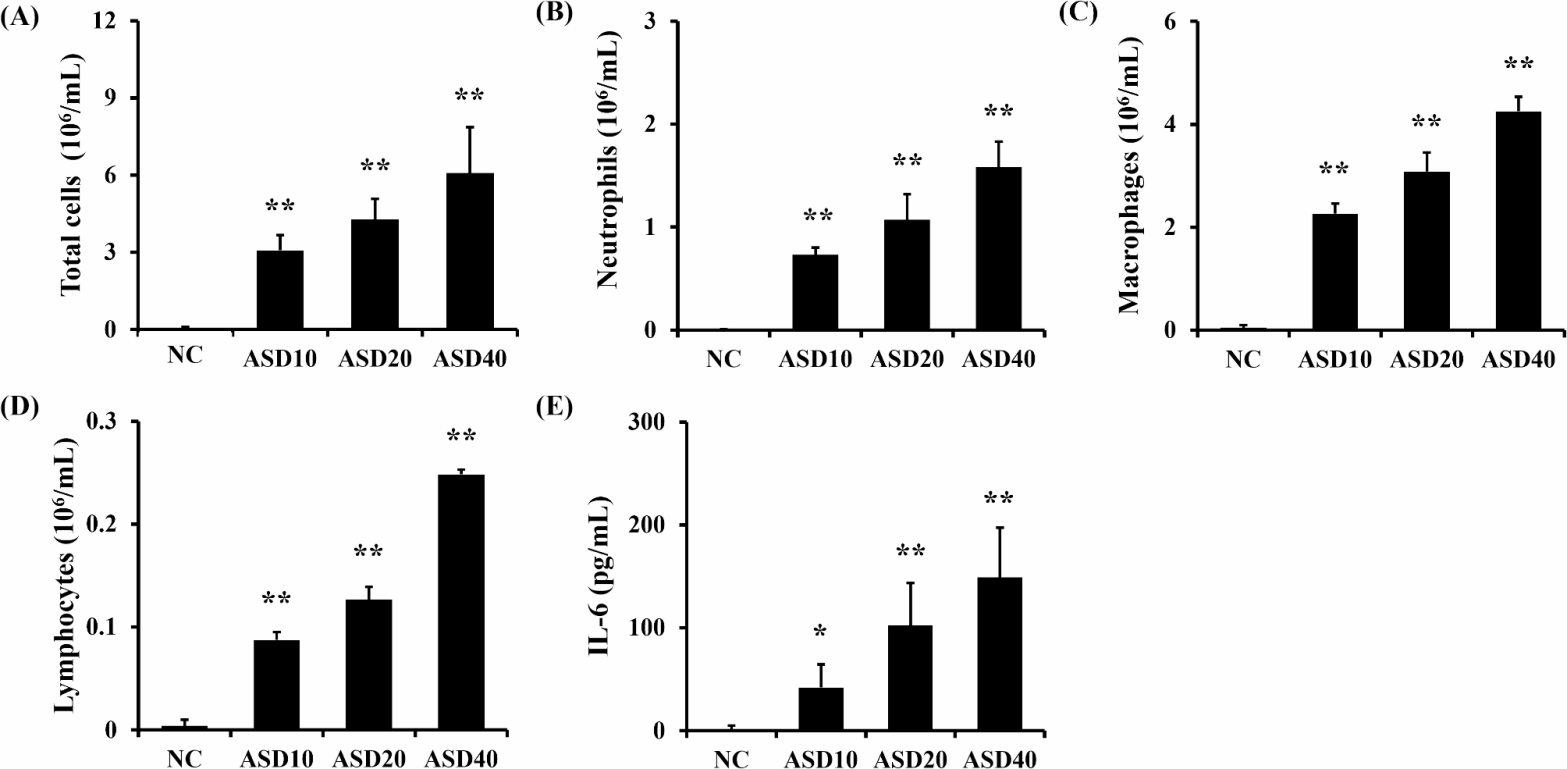
Effects of ASD on inflammatory cell counts and inflammatory cytokines in Bronchoalveolar lavage fluid (BALF). **(A)** Total cells, **(B)** macrophages, **(C)** neutrophils, **(D)** lymphocytes, and **(E)** Interleukin (IL)-6 levels. Normal control (NC), PBS intranasal instillation: ASD10, 20, and 40, ASD (10, 20, and 40 mg/kg, respectively) intranasal instillation (*n* = 7). ^*^,^**^, versus NC, *p* < 0.05 and < 0.01, respectively

ASD groups had significantly elevated the number of inflammatory cells in the BALF compared with the NC group, which was dose-dependent (Fig. 3A). In particular, the counts of macrophages and neutrophils in the BALF was considerably elevated in comparison to those of the NC group (Fig. 3B, C). In addition, the number of lymphocytes was markedly elevated by ASD exposure in a dose-dependent manner (Fig. 3D). The releases of IL-6 in the BALF were meaningfully increased by ASD exposure in dose-dependent manner (Fig. 3E).

### ASD exposure increased pulmonary inflammation and p-NF-κB expression in pulmonary tissues

**Fig. 4:**
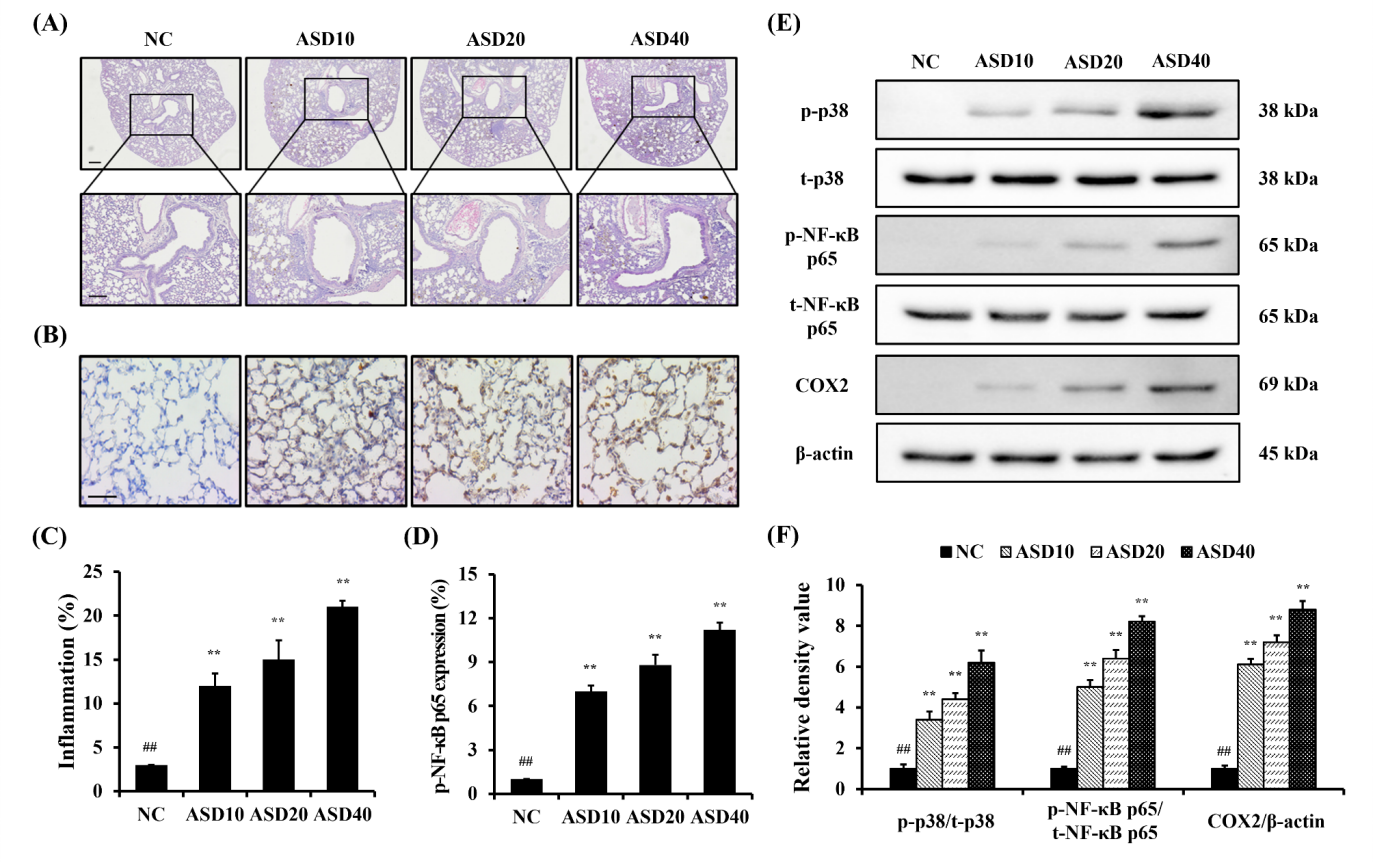
Effects of ASD on inflammatory responses and p-NF-κB expression in pulmonary tissues. **(A)** Representative figure of Hematoxylin & Eosin (H&E) staining, **(B)** representative figure for Immunohistochemical (IHC) (p-NF-κB p65), **(C)** quantitative analysis of inflammation, **(D)** quantitative analysis of p-NF-κB p65 expression, **(E)** representative figure for Western blot, **(F)** Relative density value of each protein. NC, PBS intranasal instillation; ASD10, 20, and 40, ASD (10, 20, and 40 mg/kg, respectively) intranasal instillation (*n* = 7). ^**^, versus NC, *p* < 0.01

ASD groups had a considerably increased infiltration of inflammatory cells into pulmonary tissues compared with the NC group, which was dose-dependent (Fig. 4A, C). Similar to the results of inflammatory responses in lung tissues, p-NF-κB p65 expression was markedly increased by ASD exposure, which was dose-dependent (Fig. 4B, D). Western blotting showed that ASD groups had significantly elevated expressions of p-p38, p-NF-κB p65, and COX2, which was dose-dependent (Fig. 4E, F).

### ASD exposure elevated the pathophysiological index in the BALF and serum from OVA-inhaled mice

**Fig. 5:**
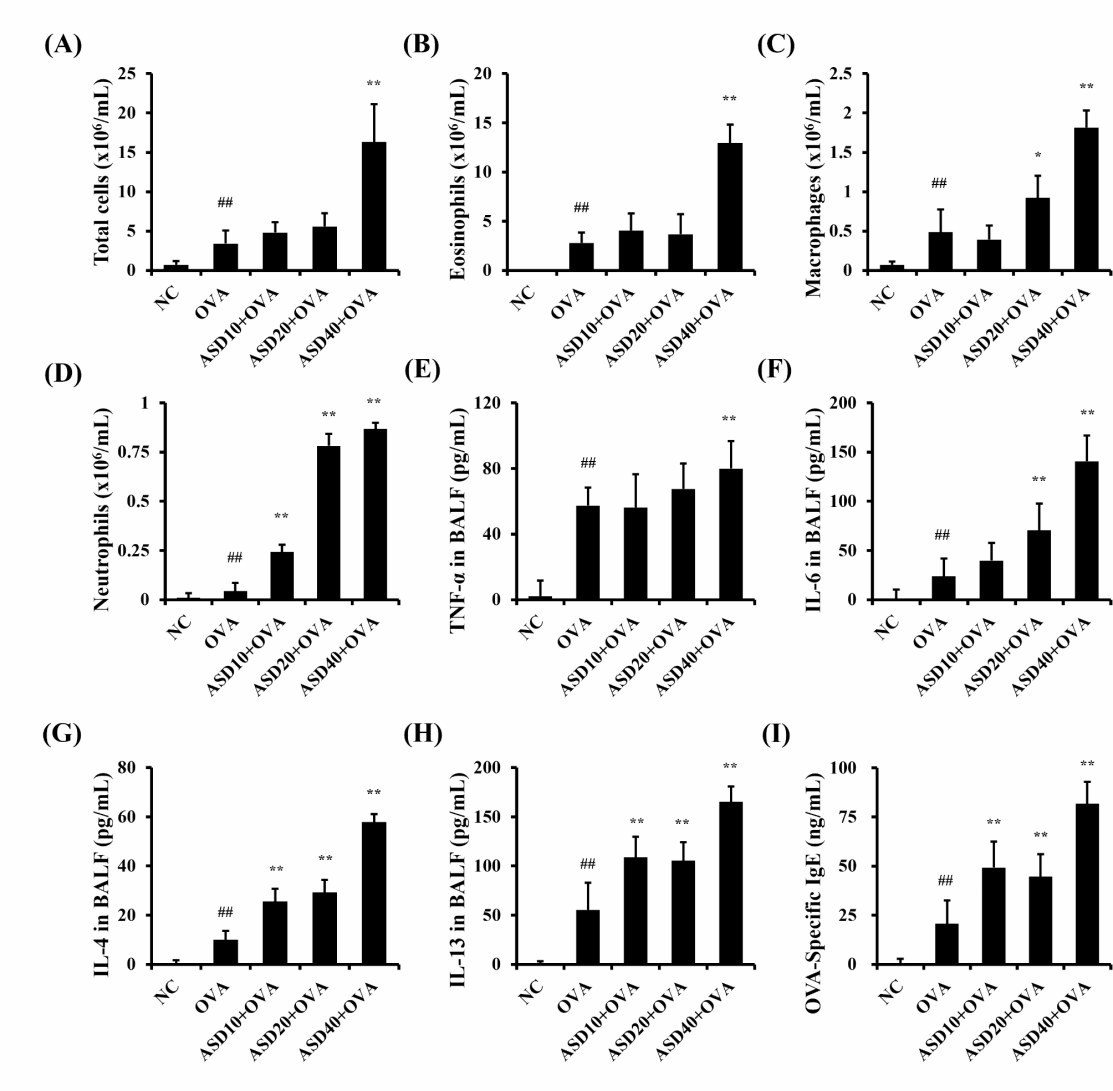
Effects of ASD on the pathophysiological indices of BALF and serum in asthmatic mice. **(A)** Total cells, **(B)** eosinophils, **(C)** macrophages, **(D)** neutrophils, **(E)** TNF-α in BALF, **(F)** IL-6 in BALF, **(G)** IL-4 in BALF, **(H)** IL-13 in BALF, and **(I)** OVA-specific immunoglobulin (Ig) E in serum. NC, normal control; ovalbumin (OVA), Asthma model + PBS intranasal instillation; OVA + ASD10, 20, and 40, Asthma model + ASD (10, 20, and 40 mg/kg, respectively) intranasal instillation (*n* = 7). ^##^, versus NC, *p* < 0.01. ^*^,^**^, versus OVA, *p* < 0.05 and < 0.01, respectively

The OVA group had a considerably elevated the number of inflammatory cells in the BALF compared with those of the NC group (Fig. 5A–E). However, ASD + OVA groups elevated the number of inflammatory cells in the BALF compared with those of the OVA group according to the increase in dose of ASD, and significant differences were detected in the ASD40 + OVA group.

The releases of TNF-α, IL-4, -6, and − 13 were obviously elevated in the OVA group in comparison to those of the NC group (Fig. 5F–I). ASD + OVA group increased inflammatory cytokines in the BALF compared with those of the OVA group, and considerable differences in the releases of IL-4, -6, and − 13 were seen in the ASD + OVA groups. Consistent with the results of cytokines, the release of OVA-specific IgE was considerably elevated in the OVA group in comparison to the NC group. Moreover, ASD + OVA group significantly elevated in comparison to the OVA group (Fig. 5J).

### ASD exposure increased AHR and mucus production in OVA-inhaled mice

**Fig. 6:**
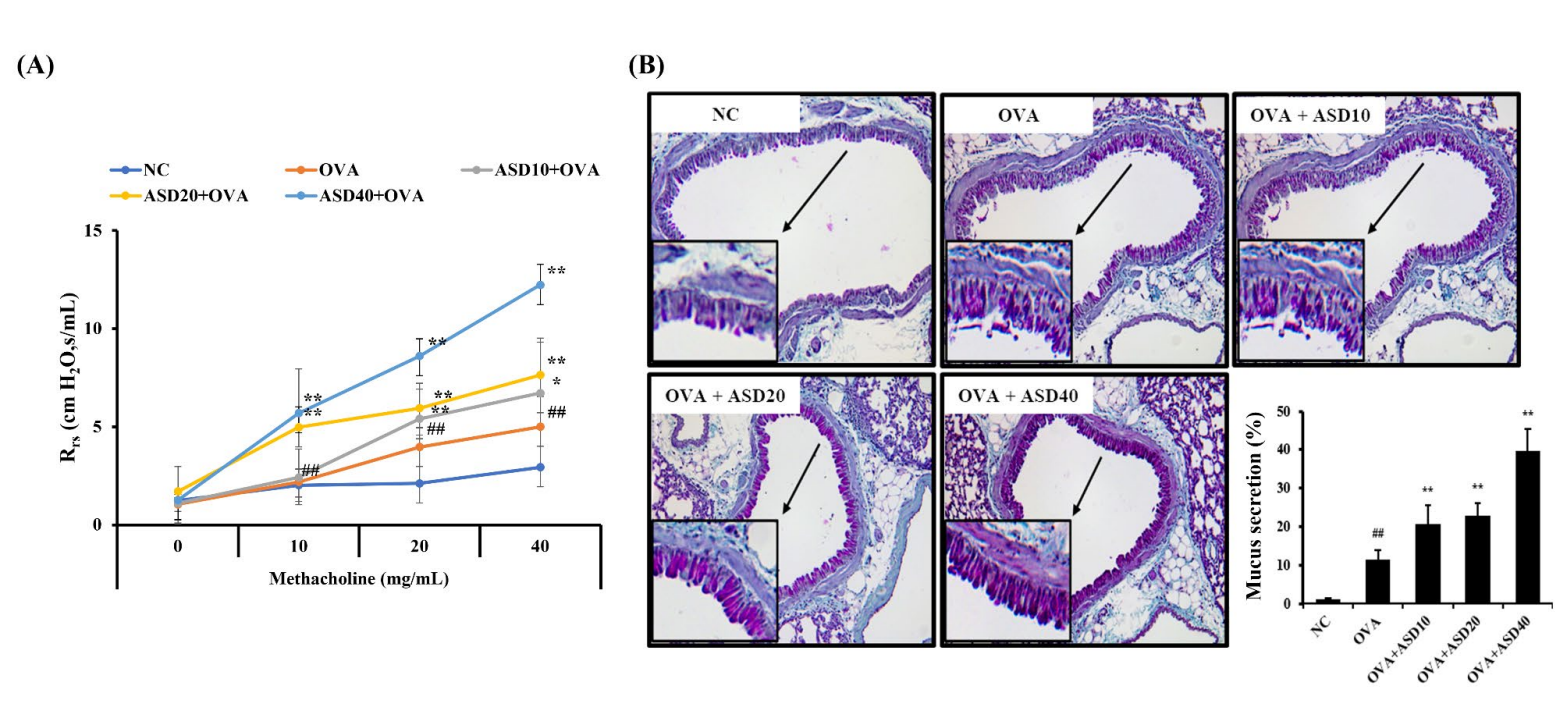
Effects of ASD on airway hyperresponsiveness (AHR) and mucus production in asthmatic mice. **(A)** Airway hyperresponsiveness, **(B)** figure of mucus production and quantitative analysis. NC, normal control; OVA, Asthma model + PBS intranasal instillation; OVA + ASD10, 20, and 40, Asthma model + ASD (10, 20, and 40 mg/kg, respectively) intranasal instillation (*n* = 7). ^##^, versus NC, *p* < 0.01. ^**^, versus OVA, *p* < 0.01

The OVA group had markedly elevated AHR compared with the NC group along with an increasing dose of methacholine (Fig. 6A). ASD + OVA group increased in comparison to those of the OVA group, especially in the ASD40 + OVA group. In addition, the OVA group had considerably elevated mucus production compared with the NC group (Fig. 6B). ASD + OVA group considerably elevated mucus production in comparison to the OVA group.

### **ASD exposure elevated airway inflammation and p-NF-κB expression in pulmonary tissues from OVA-inhaled mice**

**Fig. 7:**
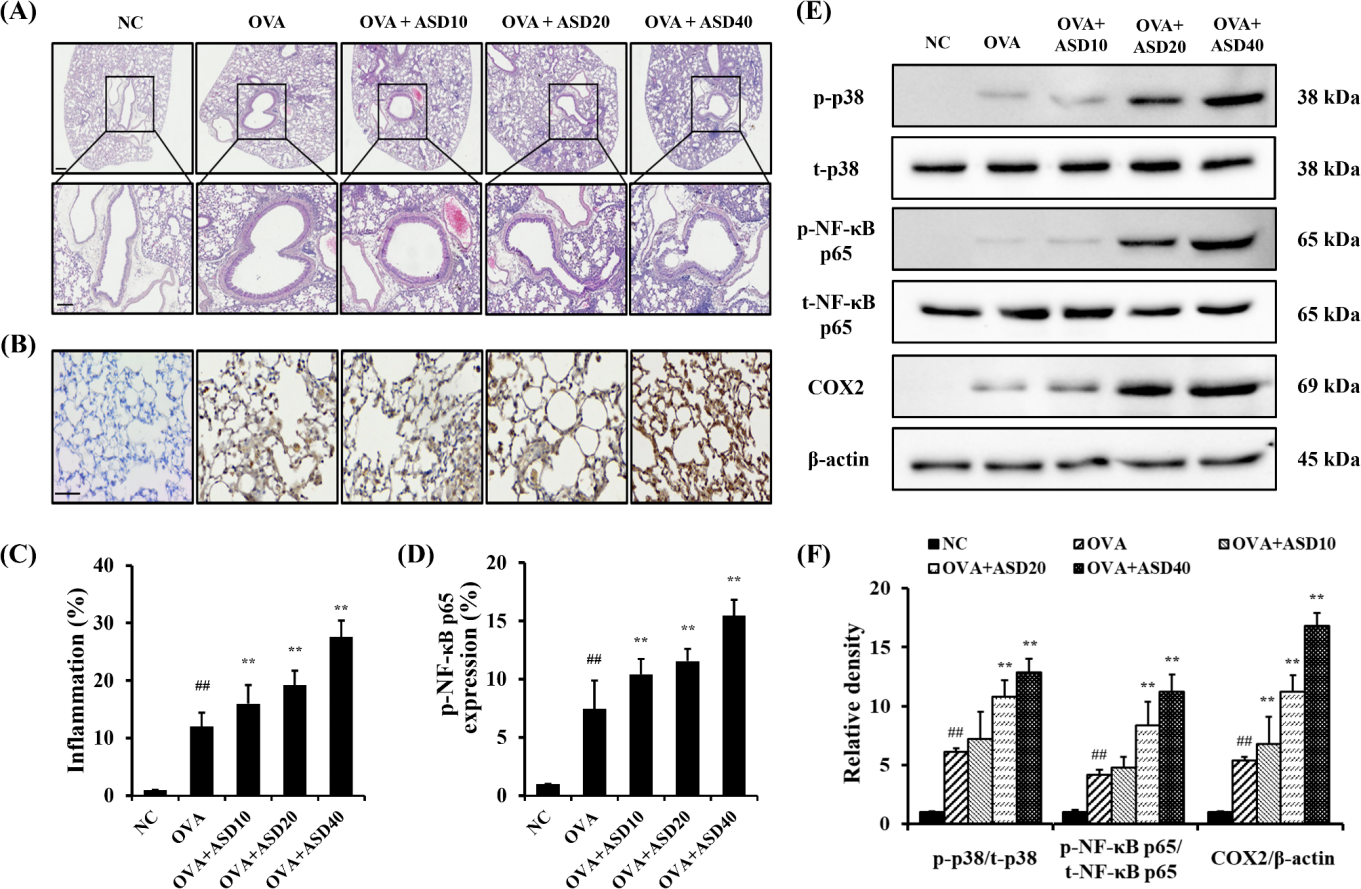
Effects of ASD on pulmonary inflammation and p-NF-κB expression in asthmatic mice. **(A)** Representative figure of H&E staining, **(B)** representative figure of IHC (p-NF-κB p65), **(C)** quantitative analysis of inflammation, **(D)** quantitative analysis of p-NF-κB p65 expression, **(E)** representative figure of Western blot, **(F)** quantitative analysis of each protein. NC, normal control; OVA, Asthma model + PBS intranasal instillation; OVA + ASD10, 20, and 40, Asthma model + ASD (10, 20, and 40 mg/kg, respectively) intranasal instillation (*n* = 7). ^##^, versus NC, *p* < 0.01. ^**^, versus OVA, *p* < 0.01

The OVA group had a markedly increased accumulation of inflammatory cells into pulmonary tissues compared with the NC group (Fig. 7A, C). ASD + OVA group considerably increased the accumulation of inflammatory cells into pulmonary tissues compared with the OVA group. Consistently, p-NF-κB p65 expression was obviously elevated in the OVA group compared with the NC group (Fig. 7B, D). ASD + OVA group considerably elevated p-NF-κB p65 expression in comparison to the OVA group. The OVA group had obviously elevated expressions of p-p38 and p-NF-κB p65 in comparison to the NC group (Fig. 7E, F). ASD + OVA group considerably elevated the expression of p-p38 and p-NF-κB p65 in comparison to the OVA group. Additionally, COX2 expression was obviously elevated in the OVA group compared with the NC group. But, ASD + OVA group increased COX2 expression compared with the OVA group.

## Discussion

The risk of ASD is increasing every year, and in particular, as various harmful components such as chemicals, nanoparticles, bacterial toxins, and allergens are included, the possibility of threatening human health is very high [17]. In Northeast Asia, where ASD mainly occurs, the prevalence of various diseases caused by ASD is continuously increasing [18]. Particularly, exposure to ASD has been involved in an elevation of the occurrence of respiratory diseases or aggravation of respiratory diseases [19]. In present study, we investigated the respiratory toxicity of ASD and explored the effects of its exposure on underlying respiratory disease using OVA-inhaled allergic asthma. The exposure to ASD markedly increased inflammatory cells and cytokines in the BALF, which was accompanied by pulmonary inflammation with the phosphorylation of NF-κB expression. In addition, ASD exposure elevated AHR, cytokines, the number of inflammatory cells, and OVA-specific IgE in OVA-inhaled mice, which was accompanied by the exacerbation of histopathological alterations of asthma including pulmonary inflammatory responses and mucus production. These responses were associated with the increase in p-NF-κB p65 expression as well as toxicity of ASD.

In this study, the particle size distribution of ASD, with a primary size of 292 ± 186.2 nm and a predominantly spherical morphology, places it within the inhalable range, facilitating its suspension in the air and potential to reach the lungs upon inhalation [20]. Particles with a diameter smaller than 10 μm can reach the lungs, and those smaller than 1 μm have the potential to enter the bloodstream, triggering immune responses. ASD consists primarily of fine and ultrafine particles, with a significant portion smaller than 2.5 μm. The elemental composition of ASD, with a high percentage of silica (43.4%) and oxygen (48.82%), is consistent with previous studies on desert dust particles, which are primarily composed of silicate minerals [21]. The presence of aluminum (4.73%) further suggests that ASD may possess oxidative properties, which could be relevant for assessing its potential toxicity and role in respiratory diseases [8]. The zeta potential of -32.48 mV indicates that the particles are stable in suspension, which is an important factor for understanding how ASD may remain airborne for extended periods, potentially increasing exposure risk. These findings align with previous research indicating that particles of similar size and composition can penetrate deeply into the respiratory system, potentially leading to chronic health effects such as inflammation and oxidative stress [22, 23].

Exposure to ASD was harmful to the respiratory tract through the enhancement of release of inflammatory cytokines [24]. In particular, IL-6, a well-known inflammatory marker, activates the immune system in response to specific irritants, leading to increased neutrophil production in the bone marrow. Consequently, exposure to ASD induces the accumulation of neutrophils at damaged lesions [25–27]. Accumulated neutrophils consistently induced the infiltration of inflammatory cells into damaged lesions via their chemoattractant property and damaged normal cellular structures via the production various proteolytic enzymes caused by degranulation [28, 29]. In the present study, exposure to ASD elevated the number of inflammatory cells and cytokines in the BALF, similar to the effects observed with exposure to silica dioxide and aluminum oxide nanoparticles [30, 31], which was confirmed by histological analysis.

Allergic asthma is a complex pulmonary inflammatory disorder that AHR, pulmonary inflammation and mucus secretion [16]. Among various inflammatory cells, eosinophils, neutrophils, and macrophages are activated by many allergens and then release inflammatory mediators by degranulation. Type 2 cytokines, including IL-4, -5, and − 13, are involved in promoting B cell class switching to produce IgE, recruiting eosinophils to the airways, stimulating goblet cell hyperplasia, and increasing airway smooth muscle contractility [32]. Recently, with an increase in air pollution, the degree of allergic asthma has increased and poses a serious threat to human health [12]. In present study, ASD exposure in OVA-inhaled mice elevated the asthmatic index including cytokines, the number of inflammatory cells, and allergen specific IgE compared with OVA-exposed mice, which is consistent with the evidence of histological examination, showing pulmonary inflammatory responses and mucus production. These results are consistent with a previous study [12]. Like the results of the toxicity study of ASD, when ASD was exposed to asthmatic animals, there was a considerable elevation in inflammatory cytokines and numbers of neutrophils and eosinophils in the BALF. In a murine model of ovalbumin-induced allergic asthma, co-exposure to silica dioxide nanoparticles and aluminum oxide nanoparticles exacerbated allergic airway inflammation associated with an increased type 2 immune response [8, 33]. These evidence indicated that ASD exposure may aggravate inflammatory responses induced by allergic asthma.

NF-κB and mitogen-activated protein kinase (MAPK) are central mediators in the progression of inflammatory responses during the progression of various diseases [11, 34]. NF-κB p65 is phosphorylated via a specific stimulus and is then translocated into the nucleus, where it eventually causes the transcription of numerous inflammatory factors such as COX2, TNF-α, IL-1β, and − 6. The p38 MAPK plyas a pivotal role in inflammation, participating in both innate and adaptive immune responses by regulating the various inflammtory mediators [35]. NF-κB recruitment is regulated by p38 MAPK by increasing the accessibility of NF-κB binding sites in NF-κB-dependent promoters [36]. It has also been shown that p38 MAPK and NF-κB regulate the level of COX2 expression in airway myocytes [37]. Due to this series of reactions, an inflammatory response develops in lesions exposed to the specific stimulus. In this study, ASD exposure induced the phosphorylation of NF-κB p65 in pulmonary tissues, which was accompanied by an increase in inflammatory cell accumulation into pulmonary tissues. This evidence indicated that pulmonary inflammatory responses caused by ASD exposure was associated with the p-NF-κB p65. Several studeis have reported that PM exposure induces airway inflammation mediated by p-NF-κB pathway [22, 38]. In addition, NF-κB p65 appears to be important in allergic asthma [13]. The expression of p-NF-κB was enhanced in bronchial biopsies and sputum from asthmatic patients and a murine model of allergic asthma, compared to controls [39]. However, NF-κB knockout mice exhibited a reduction in airway inflammation responses to allergen exposure via a decrease in the releases of cytokines and chemokines [40]. In present study, ASD exposure to asthmatic mice markedly elevated the expression of p-NF-κB p65 in pulmonary tissues compared with asthmatic mice alone, which was accompanied by the elevation of the number of inflammatory cells, cytokine, AHR, and mucus production. These results indicated that ASD exposure may exacerbate the degree of allergic asthma via the phosphorylation of NF-κB expression.

## Conclusions

Exposure to ASD leads to pulmonary inflammatory responses, including an elevation of inflammatory cell infiltration and cytokine production. These reactions were exacerbated in OVA-inhaled asthmatic mice and were involved with the phosphorylation of NF-κB expression. Therefore, our results indicated that exposure to ASD may be an important risk factor for patients with allergic asthma.

## Electronic supplementary material

Below is the link to the electronic supplementary material.

Supplementary Material 1

## Data Availability

The datasets used and/or analyzed during the current study are available from the corresponding author upon reasonable request.

## Abbreviations

AHR: Airway hyperresponsiveness
ASD: Asian sand dust
BALF: Bronchoalveolar lavage fluid
COX2: Cyclooxygenase 2
ELISA: Enzyme-linked immunosorbent assay
Ig: Immunoglobulin
IL: Interleukin
MAPK: Mitogen-activated protein kinase
NC: Normal control
NF-κB: Nuclear factor-kappa B
OVA: Ovalbumin
PBS: Phosphate-buffered saline
PM: Particulate matter
SEM: Scanning electron microscopy
TBST: Tris-buffered saline containing Tween 20
TEM: Transmission electron microscopy
TNF: Tumor necrosis factor
