# Supplementary figures and images for: Asian sand dust exacerbates airway inflammation in a mouse model of asthma
Source: Lab Anim Res. 2025 May 9;41:13. doi: 10.1186/s42826-025-00243-9 (PMC12063399; doi:10.1186/s42826-025-00243-9)

Fig. 7, p-p38

Fig. 4, p-p38

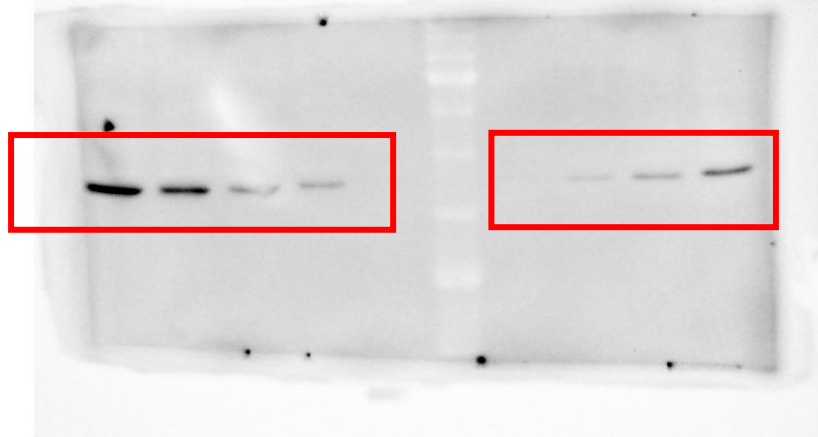

Fig. 7, p-p65

Fig. 4, p-p65

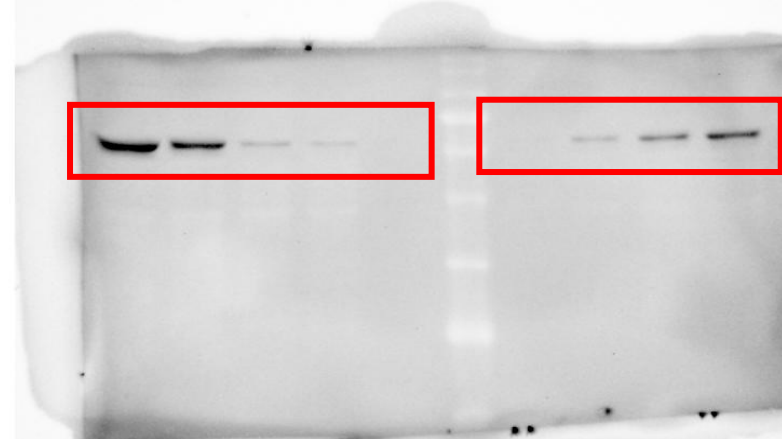

Fig. 7, COX2

Fig. 4, COX2

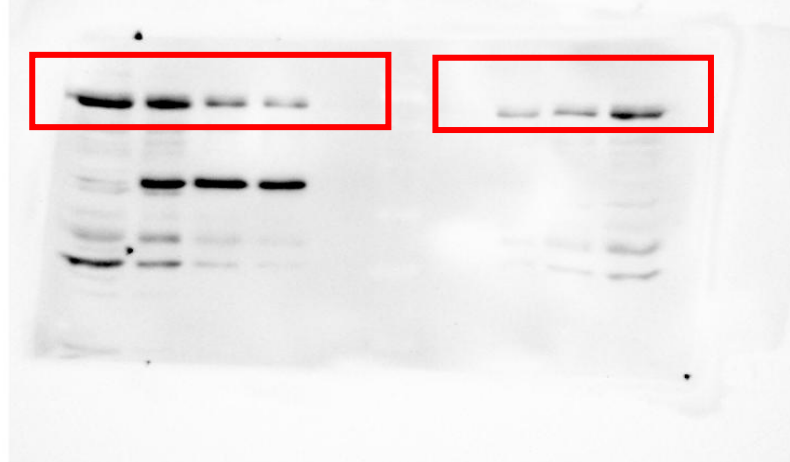

Fig. 7, Actin

Fig. 4, Actin

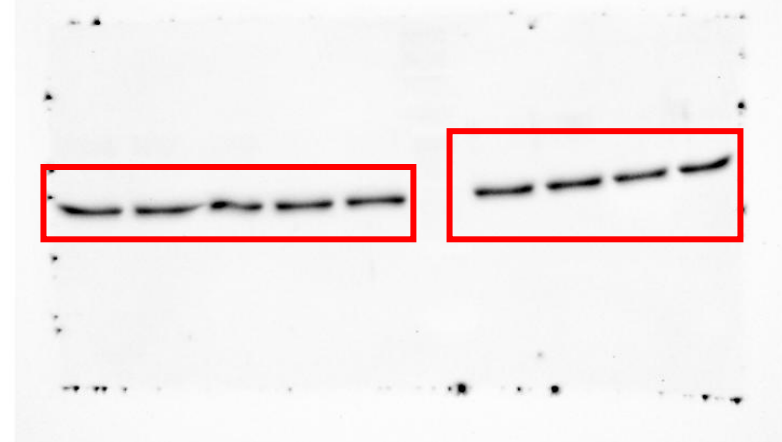

Supplement: Supplementary file 1 — Supplementary Material 1 [file 42826_2025_243_MOESM1_ESM.pdf]
